# Real-world evidence of ocrelizumab-treated relapsing multiple sclerosis cohort shows changes in progression independent of relapse activity mirroring phase 3 trials

**DOI:** 10.1038/s41598-023-40940-w

**Published:** 2023-09-11

**Authors:** J. Ingwersen, L. Masanneck, M. Pawlitzki, S. Samadzadeh, M. Weise, O. Aktas, S. G. Meuth, P. Albrecht

**Affiliations:** 1 Department of Neurology, Medical Faculty, Heinrich-Heine-University Düsseldorf, Düsseldorf, Germany; 2 Hasso Plattner Institute, University of Potsdam, Potsdam, Germany; 3 Department of Neurology, Maria Hilf Clinics, Moenchengladbach, Germany; 4 Experimental and Clinical Research Center, Charité – Universitätsmedizin Berlin, Corporate Member of Freie Universität Berlin and Humboldt-Universität zu Berlin, Berlin, Germany; 5 Department of Regional Health Research and Molecular Medicine, University of Southern Denmark, Odense, Denmark; 6 Department of Neurology, Slagelse Hospital, Slagelse, Denmark

**Keywords:** Neuroimmunology, Multiple sclerosis

## Abstract

Ocrelizumab is a B cell-depleting drug widely used in relapsing–remitting multiple sclerosis (RRMS) and primary-progressive MS. In RRMS, it is becoming increasingly apparent that accumulation of disability not only manifests as relapse-associated worsening (RAW) but also as progression independent of relapse activity (PIRA) throughout the disease course. This study’s objective was to investigate the role of PIRA in RRMS patients treated with ocrelizumab. We performed a single-center, retrospective, cross-sectional study of clinical data acquired at a German tertiary multiple sclerosis referral center from 2018 to 2022. All patients with RRMS treated with ocrelizumab for at least six months and complete datasets were analyzed. Confirmed disability accumulation (CDA) was defined as a ≥ 12-week confirmed increase from the previous expanded disability status scale (EDSS) score of ≥ 1.0 if the previous EDSS was ≤  5.5 or a ≥ 0.5-point increase if the previous EDSS was > 5.5. PIRA was defined as CDA without relapse since the last EDSS measurement and at least for the preceding 12 weeks. RAW was defined as CDA in an interval of EDSS measurements with ≥ 1 relapses. Cox proportional hazard models were used to analyze the probability of developing PIRA depending on various factors, including disease duration, previous disease-modifying treatments (DMTs), and optical coherence tomography-assessed retinal degeneration parameters. 97 patients were included in the analysis. Mean follow-up time was 29 months (range 6 to 51 months). 23.5% developed CDA under ocrelizumab therapy (n = 23). Of those, the majority developed PIRA (87.0% of CDA, n = 20) rather than RAW (13.0% of CDA, n = 3). An exploratory investigation using Cox proportional hazards ratios revealed two possible factors associated with an increased probability of developing PIRA: a shorter disease duration prior to ocrelizumab (*p* = 0.02) and a lower number of previous DMTs prior to ocrelizumab (*p* = 0.04). Our data show that in ocrelizumab-treated RRMS patients, the main driver of disability accumulation is PIRA rather than RAW. Furthermore, these real-world data show remarkable consistency with data from phase 3 randomized controlled trials of ocrelizumab in RRMS, which may increase confidence in translating results from tightly controlled RCTs into the real-world clinical setting.

Multiple sclerosis (MS) is a disease of the central nervous system in which inflammation-associated tissue damage translates into neurological deficits of the patients. Classically, the disease is categorized as *relapsing–remitting MS* (RRMS) with temporary autoimmune attacks leading to neurological deficits, which often remit over time—with or without fixed residual symptoms—and *progressive MS* with a slow but steady accumulation of neurological deficits usually without amelioration. At disease onset, most patients experience RRMS, which frequently slowly shifts into the progressive form, also called secondary progressive MS (SPMS). A minority of patients never experience relapses and directly enter the progressive form, then called primary progressive MS (PPMS). However, the distinction between RRMS and PPMS or SPMS is not always easy. Many patients that most clinicians would characterize as RRMS rather than SPMS experience low-level, non-obvious progression, which is not associated with relapses^1^. To clarify this complex terminology, new terms have been coined to distinguish the way disability accumulates in patients depending on the disease course: *relapse-associated worsening* (RAW) describes fixed neurological deficits that occurred during a relapse with an incomplete remission on the one hand and *progression independent of relapse activity* (PIRA) on the other hand^2–5^. Recent analyses from an extensive data set show that both RAW and PIRA contribute to overall disability accumulation. Interestingly, treatment with any disease-modifying agent skewed the overall disability progression towards PIRA, presumably because of prevented relapses under therapy^6^.

Ocrelizumab is a potent immunotherapy used for treatment of PPMS and RRMS. The monoclonal antibody directed against the CD20 molecule leads to lysis and depletion of B cells. In PPMS, it remains the only approved disease-modifying treatment option to this day. In RRMS, it is most commonly used as second-line therapy when first-line treatment options turn out to be insufficiently effective. However, in highly-active disease, it is also used as first-line medication. Using data from the pivotal phase 3 randomized controlled trials (RCTs) OPERA I and OPERA II^7^, Kappos et al. investigated RAW and PIRA contributions to the disability accumulation of ocrelizumab-treated vs. interferon-treated patients^4^. They found that (A) PIRA was responsible for most of the disability accumulation rather than RAW irrespective of the treatment arm (later confirmed by the findings of Lublin et al.) and (B) ocrelizumab was superior in preventing PIRA compared to interferon. However, it was less effective in preventing PIRA than RAW.

Data from RCTs remain the indispensable golden standard for proving the efficacy and safety of new drugs. Some of RCTs' most significant strengths, i.e. homogeneity of the population, tight control of per-protocol application of the drug, very close clinical follow-up, can sometimes also be a disadvantage when the study conditions do not reflect real world clinical setting well. Therefore, complementary to RCT data, so-called real-world data play an increasing role in clinicians' decision-making in general^8^ and concerning ocrelizumab in particular^9–11^. We here set out to perform a real-world data analysis of RAW and PIRA contributions to disability accumulation in ocrelizumab-treated RRMS patients from our local single-center cohort.

## Methods

### Patients

We performed a retrospective database search and chart review study at our local tertiary MS referral center at the Department of Neurology at Heinrich-Heine-University of Düsseldorf in Germany from 2018 to 2022. All relevant data were documented during routine visits into our local database and made available for scientific analysis upon written informed consent of the patient (see below). Inclusion criteria were diagnosis of RRMS according to the 2017 revised McDonald criteria^12^, 18 to 70 years of age, Expanded Disability Status Scale (EDSS) of 0 to 7.0 at the initiation of ocrelizumab, complete longitudinal data on EDSS and relapse activity of at least six months and treatment with ocrelizumab. Exclusion criteria were inability to consent and relapse activity three months prior to initiation of ocrelizumab.

### Definition of events

Confirmed disability accumulation (CDA) was defined as a disability increase from the previous assessment measured by EDSS of ≥ 1.0 points if baseline EDSS was ≤ 5.5 points or a ≥ 0.5-point increase if baseline EDSS was > 5.5 points, confirmed at a third visit after 12 or more weeks^4^. RAW events were defined as a subset of CDA events. In these, the initial disability increase from the previous assessment occurred in an interval with a relapse. As we were not interested in the maximum EDSS change during a relapse but rather in the persisting deficit remaining after a relapse, only confirmed RAW was assessed, meaning that only ≥ 12week confirmed disability progression was analyzed, and relapse-associated worsening without confirmation was not considered for the RAW or PIRA outcome. PIRA was defined as disability accumulation independent of relapses (no relapse event between the two visits), with the following modifications to ascertain event independence of relapse activity: The previous EDSS assessment served as the baseline reference (EDSS values) just like for RAW with the exception that no relapse may have occurred within 12 weeks prior to this baseline and between the two EDSS assessments. Patients without EDSS progression were classified as stable. Several patients that were classified as PIRA patients additionally experienced relapses under ocrelizumab therapy but in intervals apart from the interval classifying them as PIRA. These relapses were classified as ‘superimposed relapses” (SIR). Peripheral blood CD19^+^ cells were measured at the end of an ocrelizumab infusion cycle before the next infusion, “not detectable” corresponded to < 1 CD19^+^ cell/µl and “detectable” to ≥ 1 CD19^+^ cells/µl.

### Spectral-domain optical coherence tomography (SD-OCT)

APOSTEL reporting recommendations were applied for SD-OCT methodology and results^13^. The methods are well-established and have also been used and described elsewhere^14–16^. For macular volume scans, 61 vertical scans centered on the fovea (30° × 25°, high-speed scanning mode) were captured. In addition, 12° peripapillary, disc-centered ring scans (high-resolution scanning mode) were obtained. For SD-OCT imaging of both eyes, the SPECTRALIS OCT device (Heidelberg Engineering, Germany) with image alignment eye-tracking software system (TruTrack and Nsite analytics, Heidelberg Engineering) was used. Averaging of macular volume scans was performed from at least 14 images per OCT image and of peripapillary ring scans from 100 scans (Automatic Real Time, ART). The image quality threshold was above 20 dB. Semi-automatic segmentation with manual correction of errors using the Heidelberg Eye Explorer software (version HEYEX 1.8.6.0, Viewing Module 5.8.3.0) of the retinal layers was performed. Only scans meeting the OSCAR-IB quality control criteria were used for analysis^17^. Layer volumes of the retinal layers were measured using the mean volume of all sectors of the standard 1, 3 and 6 mm ETDRS grid in macular volume scans. OCT scans that had been performed in the 12 months prior to baseline (i.e., initiation of OCR treatment, n = 37) were used for analysis. When both eyes had been examined, the eye with the worse values was used for analysis.

### Statistical evaluation

Calculations and general statistical analysis were performed using Python 3.8.8 (Python Software Foundation, Delaware, USA) with the SciPy package version 1.7.0^18^ and the NumPy package version 1.21.0^19^, as well as GraphPad Prism version 5.01 (La Jolla, California, USA). The results were visualized using matplotlib version 3.5.0^20^. Time-to-event analysis and related statistical testing were carried out using the lifelines python package version 0.26.3 as recommended by the package's author^21^. Normal distribution of data attributes was checked using Shapiro–Wilk and D'Agostino tests. Data are presented as mean (standard deviation) or n (%). For differences in multiple groups Kruskal–Wallis test was performed with Dunn's multiple comparison test. Time-to-event outcome was defined as first occurrence of PIRA. Since PIRA was defined as an EDSS increase with confirmation by a second EDSS measurement, it is notable that the first of the two measurements with EDSS increase accounted for the event. For analysis using Cox proportional hazard models, the cohort was divided in subgroups based on the ocrelizumab treatment onset, the number of previous DMTs, whether ocrelizumab was used as first-line therapy, sex, annualized relapse rate (ARR) at baseline, EDSS at baseline and the respective quartiles of different OCT measurements (GCIPL, pRNFL, mRNFL). Where the respective variables were not explicitly used to form the groups, the Cox proportional hazard models corrected for age at ocrelizumab initiation, sex, baseline EDSS and baseline ARR.

A propensity score to characterize the baseline characteristics of different cohort subgroups was performed using the psmpy python package version 0.3.13^22^. The propensity score for the development of PIRA was calculated using logistic regression using the variables sex, EDSS at baseline, ARR at baseline and age at ocrelizumab treatment. Propensity scores between subgroups were tested for differences using a Mann–Whitney-U test. Differences were considered statistically significant with the following *p*-values: *p < 0.05, **p < 0.01, ***p < 0.001 and ****p < 0.0001.

### Ethical approval

The local ethics committee of Heinrich-Heine-University Düsseldorf approved this study (registry number 5951R, approval date 28.06.2018, and registry number 2021–1775, approval date 12.07.2022). Written informed consent was obtained from all participants in accordance with the Declaration of Helsinki.

## Results

### Cohort and patient characteristics

**Figure 1:**
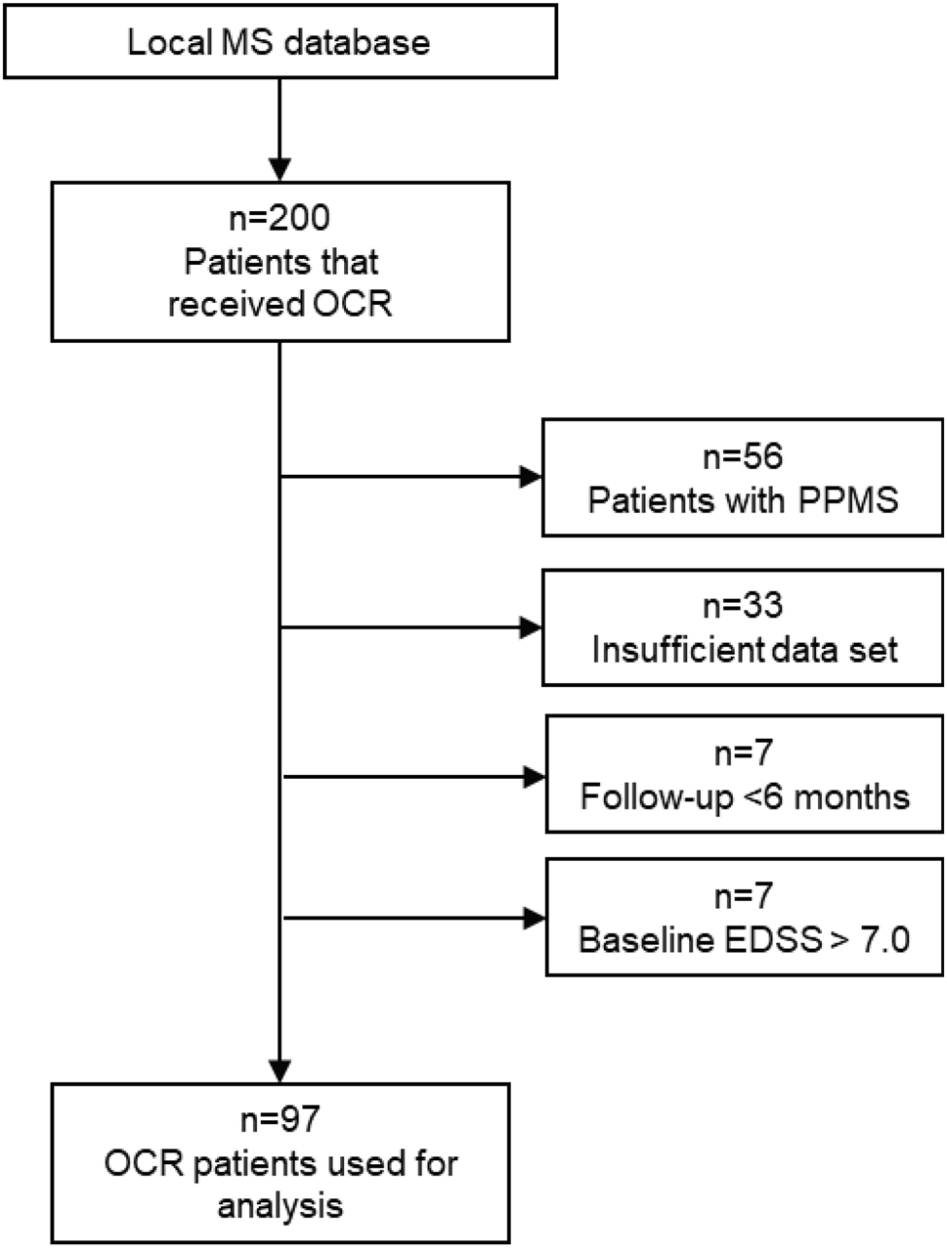
Overview of the cohort and patient selection according to inclusion and exclusion criteria. *MS* multiple sclerosis; *OCR* ocrelizumab; *PPMS* primary progressive multiple sclerosis; *EDSS* expanded disability status scale.

The flow chart in Fig. 1 shows the patient selection scheme. We searched our local database of MS patients and identified n = 200 to have received at least one dose of ocrelizumab at our local multiple sclerosis center. Of those, n = 103 were excluded: n = 56 were patients with PPMS, n = 33 had insufficient data sets, n = 7 had a too-short follow-up period of fewer than six months, and n = 7 had a baseline EDSS of > 7. n = 97 patients met all inclusion and no exclusion criteria.

Table 1 shows the demographic and clinical characteristics of these 97 patients: age at ocrelizumab initiation (i.e., baseline) was, on average, 42.3 years (± 10.7 years, median 42 years), and the sex distribution was 48.6% female. At baseline, the disease duration had been, on average, 10.5 years (± 8.5 years, median 9 years), and ocrelizumab treatment duration at the time of analysis (i.e., the follow-up time) was 28.8 months (± 12.0 months, median 31 months). The number of DMTs before ocrelizumab was, on average, 2.3 (± 1.6, median 2). The annualized relapse rate in the two years prior to baseline was 0.7 (± 0.9). EDSS at baseline was 3.5 points (± 2.2 points, median 3.5), and the overall change in EDSS in the whole cohort was an increase of 0.4 points (± 1.1 points, median 0.0).

**Table 1:** Demographical and clinical characteristics of the cohort.

Characteristics	All n = 97 (100%)	PIRA n = 20 (20.6%)	No PIRA n = 77 (79.4%)	*p*-Value
Age at OCR initiation i.e. baseline (years, mean ± SD)	42.3 ± 10.7	42.2 ± 11.1	42.4 ± 10.6	n/s (0.895)
Female sex (No, %)	48 (48.6%)	8 (42.1%)	41 (53.2%)	
Disease duration at OCR onset (years, mean ± SD)	10.5 ± 8.5	7.2 ± 6.3	11.4 ± 8.7	n/s (0.056)
OCR therapy duration (months, mean ± SD)	28.8 ± 12.0	33.8 ± 10.9	27.6 ± 12.0	n/s (0.056)
Number of DMTs prior to baseline (mean ± SD)	2.3 ± 1.6	1.7 ± 1.5	2.4 ± 1.6	n/s (0.142)
DMT treatment naïve at baseline (No, %)	18 (18.6%)	5 (25%)	13 (16.9%)	
Annualized relapse rate prior to baseline (mean ± SD)	0.7 ± 0.9	0.8 ± 1.1	0.7 ± 0.9	n/s (0.948)
EDSS at baseline (mean ± SD)	3.5 ± 2.2	3.4 ± 1.7	3.5 ± 2.3	n/s (0.336)
EDSS change during OCR treatment (mean ± SD)	0.4 ± 1.1	2.0 ± 1.0	-0.1 ± 0.5	< 0.0001

*p*-Values were calculated using Mann–Whitney U test, and *p* < 0.05 was considered statistically significant. The no-PIRA group included 71 stable patients, 3 patients with relapses with worsening, and 3 patients with relapses without worsening.

*PIRA* progression independent of relapse activity; *OCR* ocrelizumab; *SD* standard deviation; *n/s* not significant; *No* number; *DMT* disease-modifying therapy; *EDSS* expanded disability status scale.

### CDA, PIRA, and RAW in ocrelizumab-treated patients

**Figure 2:**
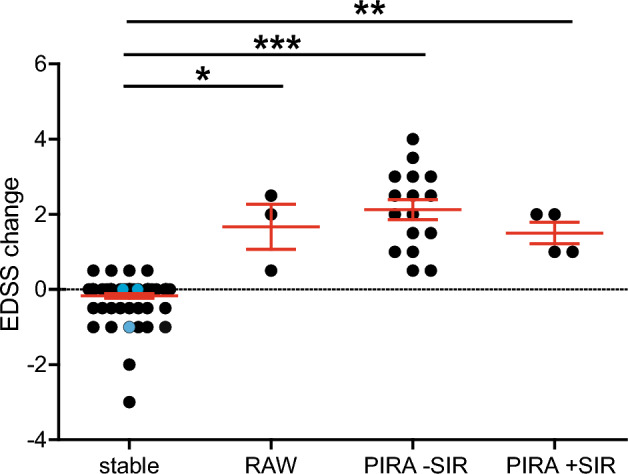
Disability change. In order to visualize EDSS changes, the total cohort (n = 97) was stratified into four groups: "stable" are patients without EDSS worsening (n = 71), "RAW" were patients with relapse-associated worsening in EDSS (n = 3), "PIRA -SIR" were patients with progression independent of relapses without superimposed relapses (n = 16), "PIRA + SIR" were PIRA patients with superimposed relapses (n = 4). There were no patients with a combination of PIRA and RAW in the cohort (i.e., no superimposed relapses altered the EDSS in any patient). n = 3 patients experienced relapses with complete remission, i.e., without detectable long-term EDSS worsening, and are depicted as blue dots within the stable group. EDSS worsening was defined as an increase of 1.0 EDSS points if the previous EDSS was ≤ 5.5 or 0.5 EDSS points if the previous EDSS was > 5.5 and if the new EDSS was confirmed at least 12 weeks thereafter. Significance was calculated using Kruskal–Wallis test and Dunn's multiple comparison test. *p < 0.05; **p < 0.01; ***p < 0.001. *EDSS* expanded disability status scale; *RAW* relapse-associated worsening; *PIRA*  progression independent of relapse activity; *SIR* superimposed relapses.

Of the 97 patients included in the analysis, 23 (23.7%) experienced CDA, i.e., a confirmed increase in EDSS according to the definition laid out in the methods section. Three patients had a RAW event (3.1% of the total population of 97 patients, three patients one relapse each), and 20 patients had a PIRA event (20.6% of the analyzed 97 patients). As depicted in Fig. 2, differences between stable patients and the other three groups were statistically significant, which is unsurprising given the fact that the presence or absence of EDSS increase defined these groups. Within the three groups with EDSS increase (RAW, PIRA -SIR, and PIRA + SIR), no statistical differences were detectable, presumably at least partly due to the small sample size. Numerically, however, we only observed relatively small differences between RAW and PIRA groups.

**Figure 3:**
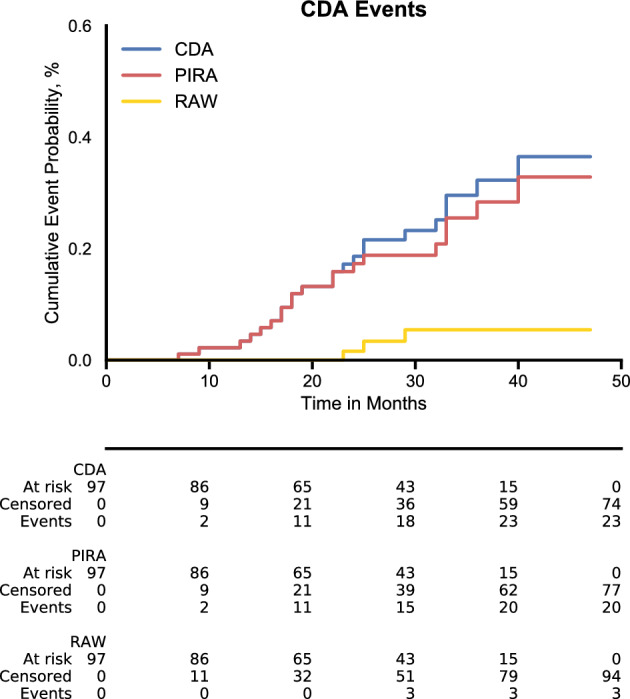
Kaplan-Meyer-estimates of CDA events in real-world ocrelizumab cohort. The figure depicts the probability of patients having experienced RAW or PIRA events in the observed cohort over time. CDA shows the probability of at least one of the two having occurred. *CDA* confirmed disability accumulation; *RAW* relapse-associated worsening; *PIRA* progression independent of relapse activity.

Figure 3 depicts a Kaplan–Meyer time-to-event analysis showing the relative contributions of RAW and PIRA to all CDA events. The figure illustrates that in the vast majority of patients, disability accumulation was due to PIRA rather than RAW.

Table 1 shows a stratification by the occurrence of PIRA and non-occurrence thereof, which showed no significant differences in patient characteristics, except for EDSS change: -0.1 points (± 0.6 points) for no-PIRA-patients and + 2.0 points (± 1.0 points) for PIRA patients (*p* < 0.0001). This result is, of course, unsurprising given the definition of PIRA.

### Investigation of factors associated with PIRA development

**Figure 4:**
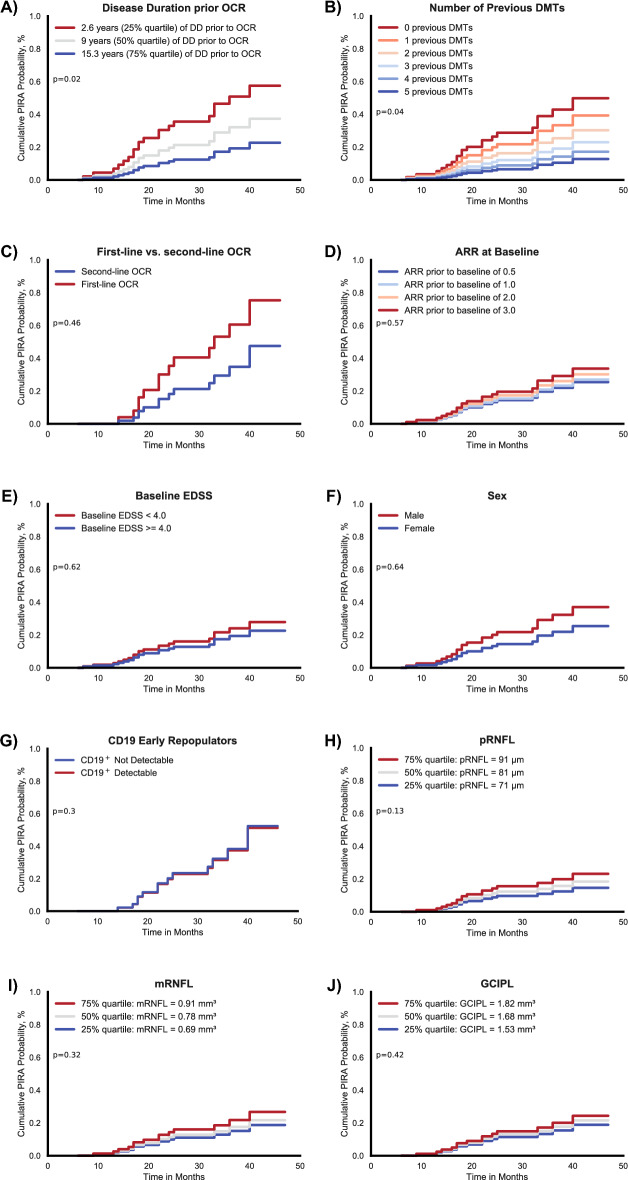
Factors associated with PIRA development probability. Kaplan-Meyer estimates of clinical, demographical, and OCT factors associated with PIRA event probabilities. The panels (**A**)–(**I**) depict the cumulative probabilities of developing PIRA according to different categories: (**A**) Disease duration prior to ocrelizumab stratified by quartiles (2.6 years, 9 years, 15.3 years). A shorter disease duration showed a significantly increased probability to developing PIRA (*p* = 0.02). (**B**) Number of DMTs used previously to ocrelizumab. Less previous DMTs was associated to a higher PIRA probability (*p* = 0.04). Other factors did not show significant alterations in PIRA probability: (**C**) Ocrelizumab as a first-line therapy vs. second-line, (**D**) ARR in the 2 years prior to ocrelizumab initiation, (**E**) EDSS at ocrelizumab initiation, (**F**) male vs. female and detectability of CD19^+^ cells in blood counts 6–12 months after OCR initiation (**G**). OCT based retinal measures were also not significantly associated with PIRA development probability with (**H**) showing peripapillary RNFL thickness, (**I**) showing macular RNFL volume and (**J**) showing macular GCIPL volume. OCT factors were stratified by quartiles with the worse of the two eyes taken into analysis. *PIRA* progression independent of relapse activity; *OCR* ocrelizumab; *DD* disease duration; *DMTs* disease-modifying therapies; *ARR* annualized relapse rate; *EDSS* expanded disability status scale; *pRNFL* peripapillary retinal nerve fiber layer; *mRNFL* macular retinal nerve fiber layer; *mGCIPL* macular ganglion cell and inner plexiform layer.

We then set out to investigate factors that could be associated with the development of PIRA. In an exploratory manner, we used Cox proportional hazard models to compare PIRA development between subgroups (Fig. 4).

In order to investigate disease duration prior to ocrelizumab therapy as a possible factor, we stratified the time since diagnosis according to quartiles, with the 25% quartile being at 2.6 years, the 50% quartile at 9 years, and the 75% quartile at 15.3 years. Figure 4A shows the differences of the cumulative probability of developing PIRA under ocrelizumab therapy between the subgroups stratified by disease duration. A shorter disease duration prior to treatment was associated with a higher probability of developing PIRA with statistical significance (*p* = 0.02). A propensity score analysis showed a higher likelihood of PIRA-risk associated baseline characteristics in patients receiving ocrelizumab after a short disease duration compared with patients that already had a long disease course (*p* = 0.04, Table 2). In line with this finding, a lower number of previous DMTs was associated with a higher probability of developing PIRA (*p* = 0.04), as shown in Fig. 4B.

**Table 2:** Distribution of baseline characteristics propensity score in different cohort subpopulations.

	Groups and Values		*p*
	PIRA	No PIRA	
Propensity Score (SD)	0.519 (0.058)	0.480 (0.082)	0.075
	Disease duration lowest quartile (≤ 2.6 years)	Disease duration highest quartile (≥ 15.3 years years)	
Propensity Score (SD)	0.527 (0.053)	0.471 (0.079)	0.038
	Number of DMTs prior to OCR lower than median (2)	Number of DMTs prior to OCR higher than median (2)	
Propensity Score (SD)	0.499 (0.071)	0.476 (0.087)	0.310
	First-line OCR	Second-line OCR	
Propensity Score (SD)	0.480 (0.084)	0.505 (0.066)	0.301
	CD19^+^ Not Detectable	CD19^+^ Detectable	
Propensity Score (SD)	0.490 (0.080)	0.472 (0.079)	0.376
	Smallest pRNFL lowest quartile (≤ 71 µm)	Smallest pRNFL highest quartile (≥ 91 µm)	
Propensity Score (SD)	0.479 (0.106)	0.502 (0.078)	0.108
	Smallest mRNFL lowest quartile(≤ 0.69 mm^3^)	Smallest mRNFL highest quartile (≥ 0.91 mm^3^)	
Propensity Score (SD)	0.453 (0.092)	0.504 (0.056)	0.142
	Smallest GCIPL lowest quartile (≤ 1.53 mm^3^)	Smallest GCIPL highest quartile (≥ 1.82 mm^3^)	
Propensity Score (SD)	0.465 (0.093)	0.510 (0.077)	0.158

A propensity score for the development of PIRA was calculated using logistic regression taking into account the baseline characteristics sex, EDSS at baseline, ARR at baseline and age at ocrelizumab treatment. A higher score indicates a higher likelihood for baseline characteristics that favor PIRA development. The table compares the scores of different subgroups. Significance of differences between subgroups was tested using a Mann–Whitney-U test. Differences were considered statistically significant with the following *p*-values: *p < 0.05, **p < 0.01, ***p < 0.001 and ****p < 0.0001.

Similarly, though not statistically significant, a numerically higher cumulative PIRA probability appeared to be present in those patients that used ocrelizumab as first-line therapy compared to those with ocrelizumab as second-line therapy, i.e. escalation therapy (Fig. 4C). PIRA probability according to ARR showed no statistical significance (Fig. 4D). EDSS at the time of ocrelizumab initiation (baseline) was stratified at 4.0 points as this cutoff marks an accepted milestone for gait disturbances. The cumulative probability of PIRA development did not differ significantly between baseline EDSS subgroups, but there was a slight numerical probability increase for less affected patients (Fig. 4E). Regarding sex (Fig. 4F), we also observed no statistically significant differences with a numerical increase of PIRA probability for the male sex. We also investigated detectability of peripheral blood CD19^+^ cells under ocrelizumab therapy and could not find an association of patients with early CD19^+^ cell repopulation and development of PIRA (Fig. 4G). Since optical coherence tomography is increasingly used as a predictive marker in MS, we performed Cox regression analyses regarding PIRA development with the most common OCT based predictors, the pRNFL (Fig. 4H), mRNFL (Fig. 4I) and mGCIPL (Fig. 4J). We stratified according to quartiles, and no statistical significance was observed in all three analyses.

Propensity score calculation based on baseline characteristics revealed no significant differences between the different patient subgroups analyzed, with the exception of EDSS based separation, for which a significant difference was observed (Table 2). Notably, the difference in propensity scores for patients with and without the occurrence of PIRA was also not significant (*p* = *0.075*).

## Discussion

In this study, we present real-world data from our multiple sclerosis center underlining that in a typical population of relapsing MS treated with ocrelizumab most of the disability worsening occurred independently of relapses (87%). Our data, therefore, add further evidence to the notion that even in typical RRMS patients, PIRA plays a relevant role in disease progression, challenging the view of a stark dichotomy between relapsing and progressive MS^1,3–5,23,24^.

Furthermore, our study also reveals the potential benefit of real-world analyses. The recent study by Kappos et al. revisited the data of the pivotal OPERA I and OPERA II trials that laid the basis for ocrelizumab's approval in RRMS by showing ocrelizumab's superiority over interferon β-1a^4^. In both, the interferon and ocrelizumab groups, PIRA (rather than RAW) was responsible for 80–90% of the clinical worsening, and ocrelizumab appeared to have a positive effect on both PIRA and RAW when compared to interferon. Our real-world data—though itself lacking a control group—corroborate the results of the Kappos study in a remarkably accurate fashion: 23.7% showed CDA (21.1% in the ocrelizumab cohort of Kappos et al.), 20.6% experienced PIRA (18.5%), and 3.1% experienced RAW (3.0%) during a mean follow-up of 29 months (21 months in Kappos et al.). This striking similarity between data from a real-world setting and from the results of a state-of-the-art phase 3 randomized controlled trial, despite obvious differences in baseline characteristics and the PIRA definition, adds confidence into both: the trustworthiness of this real-world data set on the one hand, but also the validity of results obtained from tightly controlled and arguably artificial RCTs for the real-world clinical setting.

In an exploratory manner, we investigated possible factors associated with PIRA development by Cox regression analyses. We found that a shorter disease duration prior to ocrelizumab initiation and fewer previous DMTs were associated with a higher probability of developing PIRA. Also, albeit not significant, receiving OCR as a first-line therapy appeared to be associated with higher probability to develop PIRA. Concerning biomarkers, we could not find associations with CD19^+^ cell counts. Also, no associations with OCT parameters were found, possibly due to insufficient power in light of the small differences observed in OCT and the relatively high proportion of patients that were stable.

Based on this data, one likely misguided interpretation could be that ocrelizumab is associated with PIRA development when used earlier in the disease course. In light of the solid evidence that early ocrelizumab treatment (i.e., first-line) prevents disease consistently^7^, our findings are most probably due to a confounding indication bias: patients who receive the potent disease-modifying drug ocrelizumab earlier in the disease course, thus having fewer previous DMTs, are those patients who suffer from a more aggressive disease course. This hypothesis is also supported by our exploratory propensity score analysis, which shows patients with a shorter disease course to have significantly different baseline characteristics (EDSS, ARR, sex, age) that might favor PIRA development, although the characteristics themselves did not show significant correlations with the occurrence of PIRA. Ocrelizumab may prevent some of that aggressiveness, but not all of it. As this study lacks an appropriate control, we cannot technically resolve this issue based on our data set. Since it has been suggested that not only relapses but also PIRA was prevented by ocrelizumab compared to interferon^4^, we believe the indication bias to be the best explanation for these results. Such an indication bias may also explain why Kappos et al. did not find these associations since this would not be expected in a randomized, double-blinded active-comparator trial. Furthermore, the sex distribution in our cohort was skewed towards male (51.4%) and the baseline EDSS towards higher scores (mean score of 3.5) compared to other trials such as the OPERA trials, which is probably due to an (expected) higher risk of severe disease in the male population and higher EDSS scores and thus higher probability to be put on a potent immunotherapy like ocrelizumab in a real-world setting^7^.

There are several obvious limitations to our study. The real-world data approach in itself is prone to several biases^25^. Also, the number of patients included does not allow for robust statistical calculations. Using statistical approaches such as cox regression and propensity score matching, we tried to tackle these problems, but this clearly cannot make up for a small cohort. Furthermore, the PIRA approach cannot exclude that some PIRA events are due to milder relapses that remained unnoticed or were not recalled by the patient. Also, MRI findings might have been informative, but were not conducted in a standardized way in our cohort and thus not available for analysis. Taken with the appropriate amount of precaution and considering the exploratory approach, however, we do believe that our data has value for this field of research.

Taken together, our data contributes to the notion that in typical RRMS patients under effective, immune-directed therapy PIRA is the primary driver of disability progression rather than RAW. Furthermore, given the remarkable similarity to PIRA data from the OPERA trials, our data add confidence to translating results from tightly controlled phase 3 trials into the real-world setting.

## Data Availability

Anonymized data not published within this article will be made available by request from any qualified investigator. Please direct the request at the corresponding author.
